# Decreased exposure to sunitinib due to concomitant administration of ifosfamide: results of a phase I and pharmacokinetic study on the combination of sunitinib and ifosfamide in patients with advanced solid malignancies

**DOI:** 10.1038/sj.bjc.6605696

**Published:** 2010-05-18

**Authors:** P Hamberg, N Steeghs, W J Loos, D van de Biessen, M den Hollander, M Tascilar, J Verweij, H Gelderblom, S Sleijfer

**Affiliations:** 1 Department of Medical Oncology, Erasmus University Medical Center Daniel Den Hoed Cancer Center, Rotterdam, The Netherlands; 2 Department of Medical Oncology, Leiden University Medical Center, Leiden, The Netherlands

**Keywords:** sunitinib, ifosfamide, pharmacokinetic, VEGF, drug-interaction, phase I

## Abstract

**Background:**

This study aimed to define the maximally tolerated dose (MTD) of sunitinib combined with two different infusion schedules of ifosfamide.

**Methods:**

Patients with advanced solid tumours, good performance score, good organ function, and no standard therapy available were eligible. Continuous once daily sunitinib, in escalating doses per cohort, was combined with ifosfamide, 9 g m^−2^ for 3 days or 6 g m^−2^ for 5 days, administered every 3 weeks. Pharmacokinetic (PK) and pharmacodynamic (PD) assessments were performed.

**Results:**

With growth-factor support, the MTD of sunitinib combined with either ifosfamide schedule was 12.5 mg in 32 patients enrolled. Neutropenia-related adverse events were dose-limiting toxicities. Sunitinib did not affect ifosfamide PK. Ifosfamide significantly decreased exposure to sunitinib and increased exposure to its metabolite, SU12662. No consistent changes in PD parameters were observed.

**Conclusion:**

With growth-factor support, the MTD of sunitinib with both ifosfamide schedules was 12.5 mg. Ifosfamide produced decreased sunitinib blood levels because of CYP3A induction. As PK interactions cannot explain the relatively low sunitinib doses that can be combined with ifosfamide, synergy in toxicity is likely. Whether this also holds true for anti-tumour activity needs to be further explored.

The introduction of tyrosine kinase inhibitors (TKIs) specifically inhibiting tumour-driving factors was accompanied with high expectations with regard to their activity against solid malignancies. Their single-agent activity in most tumour types is, however, modest, with obvious exceptions in tumours such as renal cell carcinoma (RCC) and gastrointestinal stromal tumours (GIST) (Verweij *et al*, 2004; Motzer *et al*, 2007).

One potential way to augment the activity of TKIs is to combine them with conventional cytotoxic agents. In particular, combinations of TKIs targeting the vascular endothelial growth factor (VEGF) pathway and conventional cytotoxic agents seem attractive, given several potential mechanisms that may yield synergistic anti-tumour effects. Vascular endothelial growth factor produced by tumour cells results in the formation of new vasculature that is abnormal in structure and more permeable than normal vasculature. This causes a high interstitial pressure within the tumour, hindering the penetration of drugs into tumours (Boucher and Jain, 1992). Inhibition of VEGF-mediated effects has been shown to decrease intra-tumoural interstitial pressure, thereby enhancing the delivery of concomitantly administered drugs (Boucher and Jain, 1992; Heldin *et al*, 2004; Willett *et al*, 2004). Other mechanisms that may contribute to synergistic interaction between VEGF-pathway inhibitors and conventional cytotoxic drugs include prevention of endothelial progenitor cell mobilisation from the bone marrow induced by chemotherapy and decreased production of tumour factors conferring resistance against chemotherapy (Simakajornboon *et al*, 2001; Dias *et al*, 2002; Tran *et al*, 2002; Riedel *et al*, 2004; Shaked *et al*, 2008).

Sunitinib is a potent inhibitor of VEGF receptors (VEGFR)1–3, KIT, platelet-derived growth factor receptor-*α* and-*β*, and Fms-like tyrosine kinase-3 (Flt3), and is one of the first and most commonly used VEGFR-TKIs. It is currently registered for the treatment of advanced RCC and imatinib-refractory GIST (Demetri *et al*, 2006; Motzer *et al*, 2007) and is being explored for its anti-tumour activity in a wide range of other tumour types. A potential attractive agent to combine with sunitinib is ifosfamide, an alkylating agent with established activity against a similar wide range of tumour entities including breast cancer, testicular cancer, lung cancer, sarcomas, and central nervous system (CNS) tumours such as medulloblastomas.

This study aimed to define the maximally tolerated dose (MTD) of ifosfamide combined with sunitinib. Two different infusion schedules of ifosfamide were explored. In addition, extensive pharmacokinetic (PK) and pharmacodynamic (PD) studies were conducted.

## Patients and methods

### Patient selection

Patients with histologically or cytologically confirmed advanced or metastatic solid tumours, for whom no standard therapy was available, with an Eastern Cooperative Oncology Group (ECOG) performance status <2, were eligible. Other inclusion criteria were evaluable or measurable disease according to RECIST version 1 (Therasse *et al*, 2000), age ⩾18 years, life expectancy ⩾12 weeks, adequate bone marrow (neutrophil count ⩾1.5 × 10^9^ cells per l; platelets ⩾100 × 10^9^ cells per l; and haemoglobin ⩾6.0 mmol l^−1^), liver (serum bilirubin ⩽1.5 × upper limit of normal (ULN) and serum ASAT and ALAT ⩽2.5 × ULN or, if liver metastases were present, ⩽5 × ULN), and renal function (serum creatinin ⩽1.5 × ULN and creatinine clearance ⩾60 ml min^−1^), two functioning kidneys, systolic blood pressure <150 mm Hg, and diastolic blood pressure <90 mm Hg (treatment with two anti-hypertensive drugs was allowed). Main exclusion criteria were history of cardiovascular disease, known HIV seropositivity, and signs or symptoms of CNS metastases.

The study was designed and conducted under the approval of appropriate institutional review boards (METC 2006–273 and CME 06–273) and in accordance with the principles embodied in the Declaration of Helsinki. Written informed consent was obtained from each participant.

### Study design and drug dosing, escalation, and administration

Daily oral sunitinib was planned to be evaluated in three dose cohorts, 12.5 mg, 25 mg, and 37.5 mg, in combination with a fixed dose of ifosfamide, according to one of the standard schedules of monotherapy ifosfamide: 9 g m^−2^ administered as 3-day continuous intravenous infusion (CIV) at 3-weekly intervals. After establishing the MTD of sunitinib with this dose and schedule of ifosfamide, this sunitinib dose was evaluated with ifosfamide at 6 g m^−2^ given as 5-day CIV. The latter ifosfamide schedule was chosen as in multidrug cytotoxic schedules; ifosfamide is frequently administered for 5 days, for example, in combination with cisplatin and etoposide. Additional patients were treated at the MTD of sunitinib with both ifosfamide regimens to get a better insight into the safety profile of the combination and to study PK drug–drug interactions. With regard to the latter, patients enrolled in these dose-expansion cohorts initiated sunitinib at day 8 of the first cycle instead of at day 1, which enables the investigation of the PK of sunitinib alone. On the basis of the mean half-life of sunitinib (40–60 h), it was anticipated that steady state of sunitinib levels was reached before initiating the second cycle of ifosfamide. Samples for PK evaluation were collected during the first two treatment cycles. After a protocol amendment because of prolonged neutropenia, granulocyte-colony stimulating factor (pegfilgrastim 6 mg once per cycle) was administered to all patients.

Twelve days before the first administration of study treatment and throughout the whole study, concurrent treatment with known CYP3A4 inhibitors or inducers was not allowed.

Using the Common Terminology Criteria for adverse events (CTCAE), version 3.0, dose-limiting toxicity (DLT) was defined as the following toxicity during the first treatment cycle: grade 4 neutropenia ⩾7 days, febrile neutropenia, grade 4 thrombocytopenia, serum creatinine ⩾2 × ULN, and any drug-related grade 3 or 4 non-haematological toxicity excluding the following events: nausea and vomiting without optimal supportive care, grade 3 fatigue <7 days, and hypertension not refractory to anti-hypertensive medication. If patients developed a systolic blood pressure >160 mm Hg, a diastolic blood pressure >100 mm Hg, or an increase in diastolic blood pressure >20 mm Hg, which (despite anti-hypertensive medication with an ACE inhibitor and a calcium-channel blocker) was not adequately controlled within 2 weeks, treatment with sunitinib was stopped. In case of grade 4 hypertension, sunitinib was also discontinued. A dose delay or interruption for longer than 2 weeks was also classified as DLT.

A classic 3+3 design was applied, implying that if a DLT was observed in one patient, three additional patients were recruited at that dose level, with the dose level escalating if no further DLT occurred at that level. If a DLT was observed in ⩾2 patients in a cohort, it could be concluded that the MTD had been exceeded. Maximally tolerated dose was defined as the highest dose level with a DLT incidence of <33%.

Before commencing each ifosfamide cycle, patients had to have neutrophils ⩾1.5 × 10^9^ cells per l and platelets ⩾100 × 10^9^ cells per l. If a patient experienced an ifosfamide-related DLT, the dose of ifosfamide was reduced by 25%. A dose reduction of more than 50% of the initial ifosfamide dose was not allowed. In those patients experiencing a DLT related to sunitinib, sunitinib was withheld for a maximum of 2 weeks. If toxicity resolved to grade 0 or 1, continuation at the next lower dose cohort level was allowed for the subsequent courses. Patients were treated for a maximum of six ifosfamide cycles. Those patients who experienced a benefit from the combination of sunitinib and ifosfamide were allowed to continue treatment with sunitinib monotherapy. Treatment was continued until disease progression or unacceptable toxicity.

### PK sampling and analysis

In patients enrolled in the expansion cohorts, blood samples for PK analysis were collected.

For ifosfamide and its most important metabolites, 2-dechloroethyl-ifosfamide, 3-dechloroethyl-ifosfamide, and 4-hydroxy-ifosfamide blood samples were collected in the presence of lithium heparin as anti-coagulant before infusion and 3, 6, 10, and 24 h after the start of ifosfamide infusion, and thereafter every 12 h until the end of infusion, before the end of infusion and 1, 3, 6, 12, and 24 h after the end of infusion during the first two treatment cycles. Blood samples were centrifuged within 15 min after collection for 10 min at 3000 **g** at 4°C. Subsequently, an aliquot of exactly 1 ml of the plasma supernatant was transferred into a vial containing 100 *μ*l of a 2 M semicarbazide solution and was stored at <−70°C until analysis of 4-hydroxy-ifosfamide. The remaining plasma was stored at <−70°C, without any additive, until the simultaneous analysis of ifosfamide and its 2-dechloroethyl and 3-dechloroethyl metabolites. Ifosfamide and the 2-dechloroethyl and 3-dechloroethyl metabolites were simultaneously quantitated by a validated liquid chromatography tandem triple quadrupole mass spectrometry (LC-MS/MS) assay. Analytes were extracted by liquid–liquid extraction from 10 *μ*l aliquots of plasma with cyclofosfamide as internal standard. For 4-hydroxy-ifosfamide, a separate LC-MS/MS method was developed and validated. Aliquots of 50 *μ*l of semicarbazide-stabilised plasma were extracted by liquid–liquid extraction with the same internal standard. Peak area ratios were a function of the concentration from 50.0 to 5000 ng ml^−1^ for all analytes, with the within and between-run precisions ⩽4.9 and ⩽5.2%, respectively, and the average accuracy ranging from 90.0 to 105.4%. Individual PK parameters for ifosfamide, 2-dechloroethyl-ifosfamide and 3-dechloroethyl-ifosfamide, and 4-hydroxy-ifosfamide were estimated using non-compartmental analysis (1/y weighting factor) using the software programme WinNonLin 5.0 (Pharsight, Mountain View, CA, USA).

For the analysis of sunitinib and its active metabolite SU12662, blood samples were taken before dosing every 3–4 days and every day during the second ifosfamide cycle. Blood samples were centrifuged within 15 min after collection for 10 min at 3000 **g** at 4°C. The plasma was stored at <−70°C, in tubes wrapped with aluminium foil, until the simultaneous analysis of sunitinib and SU12662, as recently published (De Bruijn *et al*, 2010).

### Statistical data analysis

Statistical analysis, using software package SPSS (version 15 (Softonic International, San Francisco, CA, USA)), of the changes in sunitinib concentration was carried out by Wilcoxon signed ranks test using the pre-ifosfamide sunitinib concentration as comparator. Changes in ifosfamide concentration have been evaluated using the same test. Correlation of the auto-induction rate of ifosfamide and changes in sunitinib concentrations was carried out by the Pearson's correlation test.

### Biomarker analysis

Circulating endothelial cell (CEC) enumeration, considered to reflect vascular damage, was determined using the CellSearch system (Veridex, LCC, Raritan, NJ, USA) (Rowand *et al*, 2007). Plasma concentrations of VEGF and soluble VEGFR2 (sVEGFR2) were determined using ELISA (R&D Systems, Minneapolis, MN, USA) according to the manufacturer's instructions.

## Results

### Dose escalation, MTD, and dose intensity

In total, 32 patients were enrolled (Table 1). At the first dose level (sunitinib 12.5 mg and ifosfamide 9 g m^−2^ for 3 days), a DLT occurred in two out of six patients; both experienced a prolonged, uncomplicated neutropenia (>7 days). This was pre-specified as exceeding the MTD. The protocol was amended and subsequent patients were treated with pegfilgrastim. The first dose level was repeated and no DLTs were observed. However, in the subsequent 25 mg sunitinib cohort, three DLTs occurred in five patients (two grade 4 febrile neutropenia; in one patient, hypertension accompanied by chest pain), indicating that the MTD was exceeded, rendering 12.5 mg sunitinib plus ifosfamide 9 g m^−2^ for 3 days combined with pegfilgrastim to be the MTD. After confirming the tolerability at this dose level (in total, one DLT in nine patients: grade 3 febrile neutropenia), the safety of this sunitinib dose was tested with the second ifosfamide regimen (6 g m^−2^ for 5 days), also supported with pegfilgrastim. In nine patients, one DLT was observed (grade 3 ifosfamide-induced encephalopathy).

The dose intensities of sunitinib at the MTD were 93 and 98% during combination treatment with the 3-day and 5-day schedule, respectively. A median of 4 and 3.5 cycles of ifosfamide was administered, resulting in an ifosfamide dose intensity of 93 and 96% for the 3-day and 5-day schedule, respectively.

### Toxicity

The non-haematological toxicity of sunitinib and ifosfamide was mainly grade 1–2 toxicity. Haematological toxicity was more pronounced, as grade 3–4 neutropenia was observed in 19 patients (59%), resulting in one or more episodes of febrile neutropenia in 7 patients (22%) (Table 2). At the MTD, in almost every patient in the 3-day schedule (eight out of nine; 89%), grade 3–4 neutropenia, and in four patients febrile neutropenia, occurred during combination therapy, whereas only three out nine (33%) patients treated with the 5-day schedule had grade 3–4 neutropenia, and no episodes of febrile neutropenia were observed.

### Pharmacokinetics

**Figure 1:**
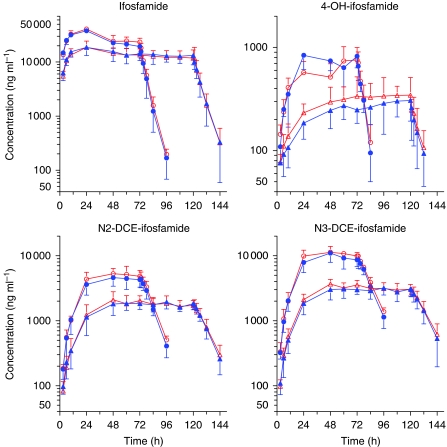
Mean concentrations (plus s.d.) of ifosfamide, 4-hydroxy-ifosfamide (4-OH-ifosfamide), 2-dechloroethyl-ifosfamide (N2-DCE-ifosfamide), and 3-dechloroethyl-ifosfamide (N3-DCE-ifosfamide) administered alone (open red symbols: bars up) or in combination with sunitinib (closed blue symbols, bars down) during the 3-day (open and closed circles) and 5-day (open and closed triangles) continuous infusions (The colour reproduction of this figure is available on the html full text version of the manuscript).

Pharmacokinetic data of sunitinib and ifosfamide were obtained in six patients per ifosfamide schedule at the MTD. Plasma concentration time curves of ifosfamide, 2-dechloroethyl-ifosfamide, 3-dechloroethyl-ifosfamide, and 4-hydroy-ifosfamide were not affected by sunitinib (Figure 1). Ifosfamide concentrations were significantly lower at 48 h (C_48h_) compared with 24 h (C_24h_) in the 3-day and 5-day schedule (40% and 17%, respectively; both *P*=0.028), in line with the known ifosfamide capacity to induce its own CYP3A-mediated metabolism (Boddy *et al*, 1995).

**Figure 2:**
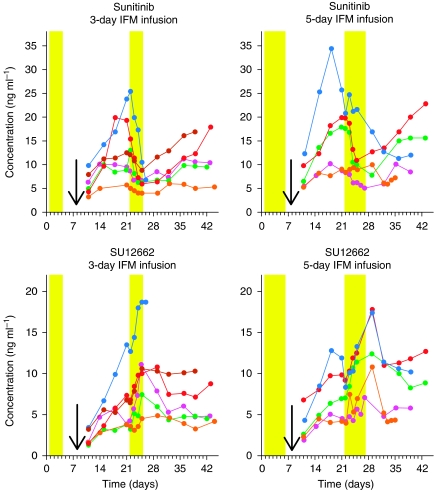
Individual plasma concentration time curves of sunitinib in combination with 3-day (*n*=6) and 5-day (*n*=5) ifosfamide schedule (upper graphs) and of SU12662 in combination with 3-day (*n*=6) and 5-day (*n*=5) ifosfamide schedule (lower graphs). One patient in the 5-day schedule has not been included in the figure as cycle two was delayed by 2 weeks. Bars represent the ifosfamide infusion schedules and arrows the first sunitinib administrations.

**Figure 3:**
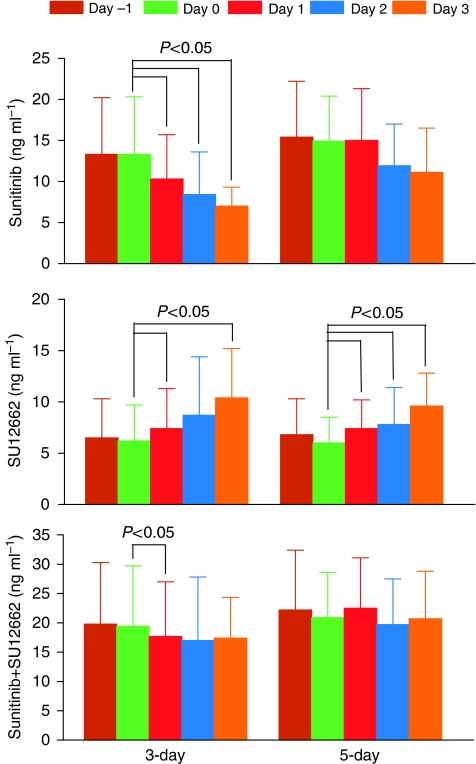
Absolute concentration of sunitinib (upper panel), SU12262 (middle panel), and the sum of sunitinib plus SU12662 (lower panel) in patients after the 3-day and 5-day continuous infusions of ifosfamide. *P*-values (Wilcoxon signed rank test) of significant lower or higher relative trough concentrations compared with the trough concentrations observed before the start of ifosfamide infusions (that is, day 0, lime bars) are presented. Day –1 (brown bars) is the sample taken the day before the start of ifosfamide infusion and days 1 (red bars), 2 (blue bars), and 3 (rose bars) are the samples taken 24, 48, and 72 h after the start of ifosfamide infusions, respectively. Data are presented as the mean±s.d. of six observations; *n*=5 for day 2 in the 3-day schedule (The colour reproduction of this figure is available on the html full text version of the manuscript).

Sunitinib trough concentrations decreased during ifosfamide infusions during the 3-day and 5-day schedule (Figure 2 upper graphs). As presented in Figure 3 (upper panel), trough concentrations were significantly lower at 1, 2, and 3 days (*P*<0.05) after the start of ifosfamide infusion in the 3-day schedule, whereas during the 5-day schedule, the decrease was less pronounced, not reaching statistical significance. The decrease in sunitinib was paralleled by an increase in the trough concentrations of its pharmacologically active metabolite SU12662 (Figure 2, lower graphs), which reached statistical significance in both the 3-day and 5-day ifosfamide schedules (Figure 3, middle panel). However, the sum of sunitinib and its pharmacologically active metabolite, SU12662, slightly decreased during the ifosfamide infusions, reaching significance only 1 day after the start of ifosfamide infusion in the 3-day schedule (*P*<0.05) (Figure 3, lower panel).

**Figure 4:**
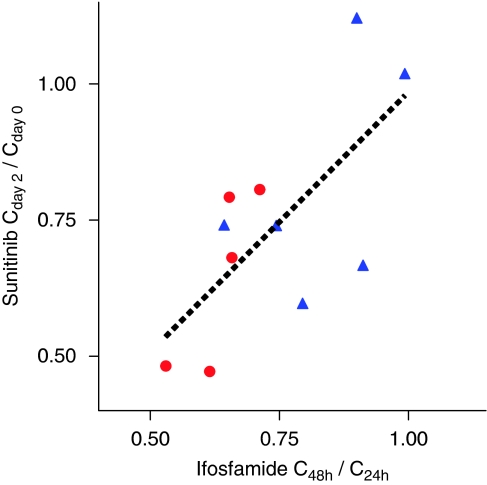
Linear relationship (*R*^2^=0.47; *P*=0.019) between auto-induction of ifosfamide and decrease in sunitinib trough concentrations during the 3-day (closed red circles) and 5-day (closed blue triangles) ifosfamide infusions (The colour reproduction of this figure is available on the html full text version of the manuscript).

The auto-induction rate of ifosfamide, expressed as C_48h_/C_24h_, was correlated to the decrease in sunitinib trough concentrations, expressed as C_day2_/C_day0_ (*R*^2^=0.47; *P*=0.019) (Figure 4).

### Biomarker analysis

Circulating endothelial cell enumeration and determination of plasma concentrations of VEGF and sVEGFR2 with serial sampling were successful in 22 patients. A wide range in relative changes in the number of CECs from baseline to post-cycle 2 (0.05–35.2) and post-cycle 5 (0.09–5.2) was observed. Relative changes from baseline to post-cycle 2 and post-cycle 5 were widespread for VEGF (0.04–14.1 and 0.25–5.6, respectively) and less outspoken for sVEGFR2 (0.63–1.4 and 0.46–1.1, respectively). No consistent change in time, correlation with dose levels, with the occurrence of febrile neutropenia, or tumour response was observed for VEGF or sVEGFR2 plasma concentrations or number of CECs.

### Anti-tumour activity

All 32 patients were assessable for efficacy. Partial responses were seen during the 3-day schedule in three patients (one patient with adenocarcinoma of unknown primary, one patient with a sarcoma NOS, and one patient with a liposarcoma). Disease stabilisation was observed in 17 patients in whom a prolonged (>3 months) disease stabilisation was observed in 9 patients (7 out of 23 patients during the 3-day schedule and 2 out of 9 patients during the 5-day schedule). The latter group consisted of patients with uveal melanoma, chordoma, chondrosarcoma, Ewing sarcoma, uterus sarcoma, endometrial carcinoma, small-cell neuroendocrine carcinoma, non-small-cell lung cancer, and carcinoma of unknown primary (all entities *n*=1).

## Discussion

This is one of the first full reports on a combination of sunitinib with a conventional cytotoxic agent and the first on the combination of ifosfamide with a VEGFR-inhibiting TKI. Combined with ifosfamide at 9 g m^−2^ given continuously over 3 days, it seemed that the MTD of sunitinib is mainly determined by neutropenia-related events. With growth factor support, sunitinib at the lowest evaluated dose of 12.5 mg daily was feasible in combination with ifosfamide at 9 g m^−2^ for 3 days and at 6 g m^−2^ for 5 days. Importantly, our data on the occurrence of neutropenia-related adverse events should be appreciated in the context of toxicity from ifosfamide monotherapy. Ifosfamide given at 9 g m^−2^ for 3 days, as an established treatment schedule, has an observed incidence in a phase III trial of 62.7% grade 3–4 neutropenia when given as first-line therapy. In addition, during a mean number 3.7 cycles of therapy, 19.6% of the patients encountered febrile neutropenia (Lorigan *et al*, 2007). Furthermore, sunitinib administered as a single agent produces grade 3–4 neutropenia in 12% of patients (Motzer *et al*, 2007). Given these figures, it comes as no surprise that neutropenia-related adverse events were the determining toxicity for the MTD of the combination. On the basis of the findings in this study, it is clear that the occurrence of neutropenia-related events is not attributable to changes in drug exposure.

Recently, several trials on sunitinib combined with conventional cytotoxic agents have been reported, although only in abstract form. The majority of the tested combinations also yielded a high incidence of neutropenia-related events, which were frequently dose-limiting toxicities. This held true for the combinations of sunitinib with docetaxel, irinotecan, FOLFIRI, carboplatin/paclitaxel, or gemcitabine/cisplatin, preventing sunitinib from being administered at full single-agent doses (Reck *et al*, 2007; Robert *et al*, 2007; Boven *et al*, 2008; Mariani *et al*, 2008; Starling *et al*, 2008; Cardoso *et al*, 2009; Friedman *et al*, 2009; Heath *et al*, 2009; Zurita *et al*, 2009). The only two combinations not hampered by neutropenia-related adverse events were sunitinib with capecitabine or with gemcitabine (Michaelson *et al*, 2008; Royce *et al*, 2008).

What becomes apparent from these studies is the fact that the schedule in which sunitinib is administered is likely to largely impact the tolerability of combinations in terms of neutropenia-related events. The recommended dose of sunitinib as a single agent was initially reported as 50 mg administered daily for 28 days every 6 weeks. However, we choose to administer sunitinib continuously. Evidence accumulates that persistent inhibition of the VEGF pathway might be advantageous over intermittent dosing, although both dosing schedules have not been directly compared yet. For combinations, however, toxicity might be more pronounced using sunitinib continuously rather than intermittently, as recovery from toxicity induced by the cytotoxic agent might be hampered in the presence of sunitinib. Accordingly, preliminary data on the combination of sunitinib with FOLFIRI suggest that continuous dosing of sunitinib was not feasible because of neutropenia, whereas in contrast, sunitinib administered in a 4-week on, 2-week off schedule could be applied at a dose of 37.5 mg in combination with FOLFIRI (Starling *et al*, 2008). From a mechanism of action point of view, the least-desirable administration schedule of sunitinib might be best combinable with chemotherapy. In contrast, sunitinib continuously administered, as well as administered in the 4-week on 2-week off schedule in combination with capecitabine, was tolerated at the same sunitinib dose (37.5 mg per day); however, as mentioned before, neutropenia was not a major issue in that specific combination (Royce *et al*, 2008).

Besides the neutropenia-related adverse events, the combination of (a relatively low dose of) sunitinib, using continuous administration, and ifosfamide was well tolerated (Table 2).

Assessing the PK of both drugs, a clear influence of ifosfamide on the PK of sunitinib was observed. This interaction resulted in a decreased systemic exposure to sunitinib and an increased exposure to its active metabolite, SU12662. Although the exact contribution of the active metabolite SU12662 to the toxicity and efficacy pattern of sunitinib in humans is unknown, preclinical data point towards equipotent inhibitory capacities (Baratte *et al*, 2004). Ifosfamide is a potent inhibitor of CYP3A, the enzyme mainly responsible for conversion of sunitinib into SU12662 (Faivre *et al*, 2006). This observed drug–drug interaction is in line with findings in healthy subjects showing a decreased systemic exposure to sunitinib when concomitantly treated with the potent CYP3A inducer rifampicin (Bello *et al*, 2005) and an increased exposure in the presence of ketoconazole, a potent CYP3A inhibitor (Washington *et al*, 2003). Pharmacokinetic drug–drug interactions have been studied combining sunitinib with gemcitabine, capecitabine, paclitaxel, and docetaxel with or without trastuzumab, but no relevant PK interactions were observed in these studies (Mariani *et al*, 2008; Michaelson *et al*, 2008; Royce *et al*, 2008; Cardoso *et al*, 2009).

As a relatively low dose of sunitinib in combination with a standard dose of ifosfamide already results in DLT, which cannot be explained by PK interactions, synergy in toxicity seems most likely. An explanatory hypothesis for the occurrence of the described neutropenia-related events while combining ifosfamide with sunitinib might consist of a dual hit. Initially, neutrophils decrease because of the administration of ifosfamide. The physiological response of mobilisation and proliferation of haematopoietic progenitor cells to restore the neutrophil count is hampered because of inhibition by sunitinib of tyrosine kinases involved in haematopoietic progenitor cell survival and proliferation, such as Flt3 and colony-stimulating factor receptor (CSF-1R).

Importantly, a multiple drug combination should not be discarded as a result of its low combinability because of synergy in toxicity alone, as this synergistic interaction may also occur at tumour cell level.

As PD parameters, CEC numbers and plasma concentrations of VEGF and sVEGFR2 were assessed. Alterations in CEC numbers is likely to reflect vascular damage (Strijbos *et al*, 2008), but no consistent changes in CEC numbers could be seen, either during therapy or between the two sunitinib doses explored. In contrast to monotherapy with sunitinib (Deprimo *et al*, 2007), no consistent pattern in the changes in plasma concentrations of VEGF and sVEGFR2 was observed.

In conclusion, the MTD of daily sunitinib combined with continuously infused ifosfamide (9 g m^−2^ for 3 days) supported by pegfilgrastim is 12.5 mg. The same dose of sunitinib is feasible using an ifosfamide schedule of 5 days (total dose per cycle 6 g m^−2^). Concomitant treatment with ifosfamide significantly decreased the systemic exposure to sunitinib, whereas the exposure to its active metabolite, SU12662, increased because of CYP3A induction. As PK interactions cannot explain the fact that ifosfamide can be combined safely only with relatively low sunitinib doses, synergy in toxicity is likely. Whether this holds true for anti-tumour activity needs to be determined, and it is particularly attractive to explore this further in tumour types against which both sunitinib and ifosfamide as monotherapy exhibit anti-tumour activity, such as soft tissue sarcomas, as well as lung and breast cancer.

## Figures and Tables

**Table 1:** Demographics and baseline characteristics (in numbers (%) if not otherwise specified)

Age (median, years)	53 (range 29–74)
	
*Gender*	
Male	16 (50)
Female	16 (50)
	
*WHO performance status*	
0	11 (34)
1	21 (66)
	
*Tumour type*	
Sarcoma	15 (47)
Chondrosarcoma	2 (6)
Leiomyosarcoma	2 (6)
Liposarcoma	2 (6)
Ewing sarcoma	2 (6)
Pleomorphic sarcoma	2 (6)
Other sarcoma types	5 (16)
Carcinoma of unknown primary tumour	3 (9)
Neuroendocrine carcinoma	2 (6)
Non-small-cell lung cancer	2 (6)
Melanoma (uveal and mucosal)	2 (6)
Miscellaneous^a^	8 (26)
	
*Previous non-hormonal systemic anticancer treatment*	
0	5 (16)
1	18 (56)
2	5 (16)
3	2 (6)
4	2 (6)

Abbreviation: WHO=World Health Organisation.

a Bile duct carcinoma, breast cancer, adenoid cystic carcinoma, endometrial cancer, urothelial cancer, pancreatic cancer, chordoma and cervical carcinoma; for all the cases *n*=1.

**Table 2:** Adverse events during combination therapy of sunitinib and ifosfamide (number of patients (%))

	**First cycle adverse events**		**Adverse events at all combination cycles**	
	**All grades**	**Grade 3/4**	**All grades**	**Grade 3/4**
*Haematological toxicity*				
Anaemia	28	0	31	2
Thrombocytopenia	19	4	22	9
Neutropenia	14	10	21	19
Febrile neutropenia	3	3	7	7
				
*Non-haematological toxicity*				
Fatigue	24	2	29	4
Nausea	20	0	24	0
Constipation	20	0	24	0
ALAT and/or ASAT	20	0	21	0
Alopecia	19	0	26	0
Vomiting	14	0	19	0
Creatinine/renal	9	0	12	1
Hypo-/hyperkalaemia	5/1	1/0	5/1	2/0
Bilirubin	5	0	6	0
Diarrhoea	4	0	5	0
Pyrosis	3	0	5	0
TSH increase/decrease	2/2	0	2/2	0
Neurotoxicity	2	1	8	1
Anorexia	2	0	11	1
Haemorrhage	1	1	1	1

Abbreviations: ALAT=alanine transaminase; ASAT=aspartate aminotransferase; TSH=thyroid-stimulating hormone.
